# Comparative efficacy of antibody-drug conjugates and chemotherapy for malignant tumors: a systematic review and meta-analysis

**DOI:** 10.3389/fonc.2025.1697340

**Published:** 2026-01-09

**Authors:** Xin Gao, Linghui Tao, Wen Hao, Xiaqiu Wu, Feiye Zhu, Shengxia Lv, Yongsheng Zhang

**Affiliations:** 1 School of Basic Medical Sciences, Zhejiang Chinese Medical University, Hangzhou, China; 2 Lanxi Hospital of Traditional Chinese Medicine, Jinhua, China; 3 School of Public Health, Zhejiang Chinese Medical University, Hangzhou, China

**Keywords:** antibody-drug conjugate, chemotherapy, malignant tumors, systematic review, meta-analysis

## Abstract

**Background:**

Despite advancements in cancer treatment, malignant tumors remain a significant global health challenge. Drawbacks of chemotherapy, such as drug resistance and strong side effects, have prompted the exploration of antibody-drug conjugates (ADCs), which combine targeting capabilities with potent cytotoxins to enhance therapeutic efficacy.

**Methods:**

We searched PubMed, Cochrane, and EMBASE library databases up to June 8, 2025, for eligible randomized controlled trials (RCTs) and extracted relevant data. The primary outcome measures were overall survival (OS) and progression-free survival (PFS), with subgroup analysis and sensitivity analysis conducted to assess the heterogeneity of statistical results.

**Results:**

A total of thirteen RCTs involving 5,927 patients were included. The results indicated that ADCs offered superior OS (HR = 0.67, 95%CI: 0.55-0.81) and PFS (HR = 0.76; 95% CI: 0.66-0.86) compared to chemotherapy drugs. The any adverse event (96.8% vs. 93.6%), grade 3–5 adverse event (51.7% vs. 51.8%) and serious adverse event (25.3% vs. 22.4%) caused to patients were generally similar between ADCs and chemotherapy.

**Conclusion:**

Our meta-analysis demonstrates that compared to chemotherapy drugs, ADC drugs can prolong both OS and PFS in cancer patients, with no significant difference in adverse reactions.

**Systematic Review Registration:**

http://www.crd.york.ac.uk/PROSPERO, identifier CRD42024592020.

## 1 Introduction

In recent years, the number of deaths caused by cancer has continued to rise. By 2024, the United States is projected to have 2,001,140 new cancer cases and 611,720 cancer deaths (1). With the continuous advancement of technology, in addition to the initial surgical interventions, radiotherapy, and chemotherapy, several new treatment modalities have gradually emerged, such as antibody-drug conjugates (ADCs) (2). ADCs are typically formed by linking monoclonal antibodies to cytotoxic drugs through linkers, enabling precise and effective eradication of cancer cells (3). The underlying principle is that the monoclonal antibody, serving as a targeted carrier for the drug, can specifically recognize and bind to specific antigens on the surface of tumor cells, and is then internalized by the tumor cells (4). Once inside, the linker is degraded by lysosomes, releasing the cytotoxin, which allows for precise destruction of the tumor cells (5).

The theoretical foundation of ADC drugs is rooted in the concept of the “magic bullet,” proposed by Paul Ehrlich, the father of immunology, in 1910 (6). In 2000, Pfizer launched the world’s first commercialized ADC drug, Gemtuzumab ozogamicin (7). Currently, ADC drugs approved by the United States Food and Drug Administration include Brentuximab vedotin (8), Trastuzumab emtansine (9), Inotuzumab ozogamicin (10), Moxetumomab pasudotox (11), Polatuzumab vedotin (12), and Enfortumab vedotin (13), among others. The mechanism of action of ADC drugs enables precise drug delivery into tumor cells, thereby enhancing the specificity and effectiveness of the therapeutic intervention (14). This implies that ADC drugs can act specifically on cancer cells without killing normal cells. Therefore, ADC drugs may have lower side effects and a potentially more powerful therapeutic efficacy (15). However, currently, ADCs are mostly used as salvage therapies, offering hope to patients with advanced tumors (16). In contrast, chemotherapeutic drugs have undergone years of clinical use and validation, leading to a mature treatment evaluation system and broader clinical application (17). The mechanism of action of chemotherapeutic drugs primarily involves killing cancer cells by interfering with their growth and division processes, including disrupting DNA synthesis, affecting RNA transcription, and protein synthesis (18). Although this approach has a broad antitumor spectrum, it also affects normal cells, leading to side effects.

In summary, ADCs and chemotherapy drugs each have their own strengths in the field of oncology treatment. ADCs can precisely target and attack tumor cells, but they also present some side effects that require further improvement (19). Although chemotherapy drugs have many side effects and issues with drug resistance, they still hold an irreplaceable position in cancer treatment (20). However, there are scarcely any meta-analyses evaluating the therapeutic effects of ADCs versus chemotherapy drugs on cancer patients. Compared to chemotherapy, can ADCs improve the clinical efficacy for patients and provide greater benefits to clinical populations? Therefore, we reviewed relevant clinical randomized controlled trials and conducted this meta-analysis.

## 2 Methods

This study was conducted according to Systematic Review and Meta-analysis (PRISMA) (21). The research question and eligibility criteria were defined *a priori* according to the PICO framework and documented in a protocol registered in PROSPERO (CRD42024592020), and the full text can be found on the website (http://www.crd.york.ac.uk/PROSPERO).

### 2.1 Search strategy

Two investigators (XG and LHT) used PubMed, Cochrane, and EMBASE library databases to search eligible studies from inception to June 8, 2025. Search terms included “Neoplasms”, “Antibody-Drug Conjugates”, “Neoplasms”, “chemotherapy” and “randomized controlled trials”, and the full electronic search strategies for each database are provided in **Supplementary Methods 1**-**3**. Moreover, the references in the relevant literature were manually reviewed to ensure that no eligible articles were missed. When different publications publish different content of the same clinical trial, the most recently published data was selected.

### 2.2 Study selection

1. Participants: patients with malignant tumor.
2. Experimental group: ADC alone, with or without placebo.
3. Control group: chemotherapy alone, with or without placebo.
4. Outcome indicators: have PFS or OS based study data.
5. Research type: randomized controlled trials.
6. Sample size of more than 10 patients in each group.

### 2.3 Exclusion criteria

1. Participants: patients with non-malignant tumor.
2. Experimental group: no use of ADC drugs, or use in combination with other therapeutic drugs.
3. Control group: no use of chemotherapy, or the combination of chemotherapy drugs with other medications.
4. Outcome indicators: no PFS or OS based study data.
5. Research type: non-randomized controlled trials.
6. Sample size of less than 10 patients in each group.

### 2.4 Literature selection and data collection

Two researchers (XG and LHT) independently extracted the relevant data, and if there is any disagreement, it will be resolved through discussion and finally reach a consensus. For included trials, specific information on study name, year of publication, treatment line, tumor type, primary endpoint, number of participants, Antibody Drug Conjugates, and chemotherapy agents were extracted.

### 2.5 Quality assessment

For each included study, two investigators (XG and LHT) independently assessed the risk of bias according to the Cochrane Handbook of Systematic Reviews of Intervention Systems (version 5.1.0) (22). If there are differences of opinion, the two researchers resolve them through discussion. We conducted a sensitivity analysis and a GRADE assessment based on the statistical results. The results are presented in the **Supplementary Materials** (**Supplementary Tables S1**-**S4**), which have enhanced the reliability of the findings.

### 2.6 Statistical analysis

All data analyses were performed using RevMan software (Windows version 5.3). Data from different trials were pooled using the Mantel-Haenszel method with either a fixed- or random-effects model, selected based on the degree of statistical heterogeneity (as opposed to clinical). Heterogeneity was assessed with the Q-test and I² statistic. The fixed-effects model was used when p > 0.1 and I² < 50%; otherwise, the random-effects model was applied. For time-to-event outcomes such as OS and PFS, hazard ratios (HRs) and their 95% confidence intervals (CIs) were calculated for each study. For dichotomous variables, odds ratios (ORs) with 95% CIs were computed. A p-value of less than 0.05 was considered statistically significant for all two-sided tests. Sensitivity analysis was also performed to assess the robustness of different aspects of legal bias in each study.

## 3 Result

### 3.1 Literature screening results

**Figure 1:**
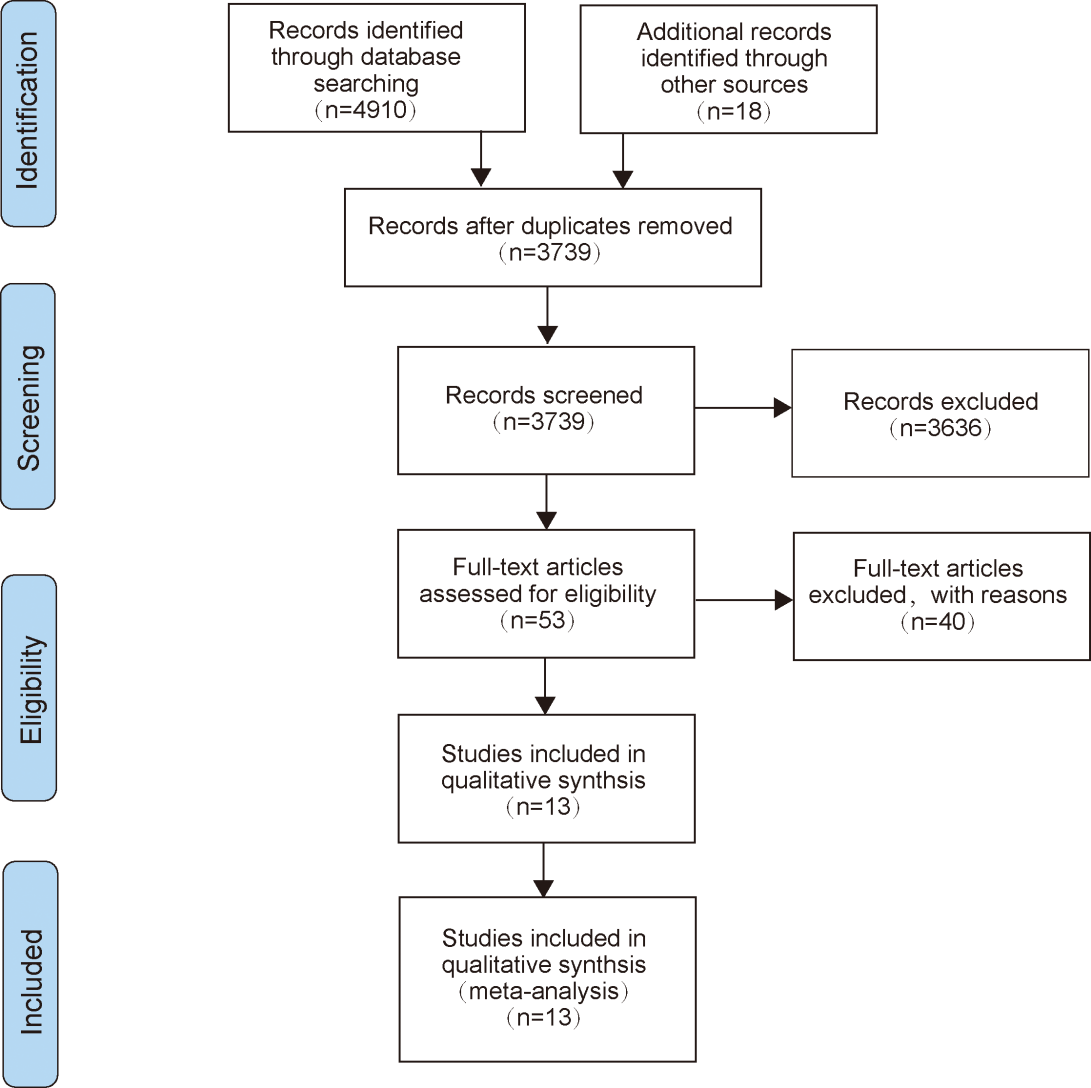
The flow diagram of literature selection.

A total of 4,910 articles were retrieved through the search strategy, and 18 related reports were retrieved manually. A total of 3,739 articles were left after the elimination of duplicates. Subsequently, we scanned the titles and abstracts, leaving us with 53 articles that met the present inclusion criteria for full-text reading. Finally, 13 of these records were included in the final analysis (23–34). The specific research inclusion process was shown in **Figure 1**.

### 3.2 Characteristics of included studies

All of the included studies were published between 2017 and 2024 and were second-line or third-line treatments (**Table 1**). Our current meta-analysis included 5,927 patients from thirteen randomized controlled trials, of which 3,392 patients were in the treatment group and 2,535 patients were in the control group.

**Table 1:** Characteristics of included studies.

Study	Year	Line	Histology	Primary endpoint	Case	Intervention arm	Control arm
ARCS-M (26)	2023	> 1	MPM	OS、PFS	166 vs. 82	AR	Vinorelbine
METRIC (32)	2021	> 1	TNBC	OS、PFS	218 vs. 109	CDX-011	Capecitabine
GATSBY (31)	2017	> 1	GC, AEG	OS、PFS	228 vs. 117	T-DM1	Taxane
MIRASOL (28)	2023	> 1	OC	OS、PFS	227 vs. 226	Elahere	Investigator’s choice
innovaTV 301 (33)	2024	> 1	CC	OS、PFS	253 vs. 249	TIVDAK	Investigator’s choice
DESTINY-Gastric01 (30)	2020	> 1	GC	OS、PFS	125 vs. 62	T-DM1	Physician’s choice
DESTINY-Breast02 (23)	2023	> 1	BC	OS、PFS	406 vs. 202	T-DM1	Physician’s choice
DESTINY-Breast04 (27)	2023	> 1	BC	OS、PFS	371vs.172	T-DM1	Physician’s choice
ASCENT (25)	2024	> 1	TNBC	OS、PFS	267 vs. 262	SG	Physician’s choice
TROPION-Breast01 (24)	2024	> 1	BC	OS、PFS	365 vs. 367	Dato-DXd	Investigator’s choice
TROPiCS-02 (34)	2023	> 1	BC	OS、PFS	272 vs. 271	SG	Physician’s choice
FORWARD I (35)	2021	> 1	OC	OS、PFS	243 vs. 109	Elahere	Stipulated chemotherapy
EV-301 (29)	2024	> 1	UC	OS、PFS	301vs.307	PADCEV	Investigator’s choice

OS, overall survival; PFS, progression-free survival; OC, ovarian cancer; BC, breast cancer; TNBC, triple-negative breast cancer; GC, gastric cancer; AEG, adenocarcinoma of esophagogastric junction; CC, cervical cancer; MPM, malignant pleural mesothelioma; UC, urothelial carcinoma; T-DM1, Trastuzumab deruxtecan; AR, Anetumab ravtansine; CDX-011, Glembatumumab vedotin; Elahere, Mirvetuximab soravtansine; SG, Sacituzumab govitecan; PADCEV, Enfortumab vedotin; TIVDAK, Tisotumab vedotin; Dato-DXd, Datopotamab deruxtecan.

### 3.3 Quality assessment

**Supplementary Figure S1** summarized the quality assessment results of the thirteen included studies. Among them, five (26–29, 33) trials were evaluated as having uncertain risk of selection bias, and eleven (23–31, 34, 35) trials were assessed as having uncertain blinding in outcome assessment. Regarding selection bias, ten (23, 24, 26, 29–35) trials were evaluated as high risk, while three (25, 27, 28) trials were assessed as having uncertain risk of bias. All others were deemed as low risk.

### 3.4 Overall survival

**Figure 2:**
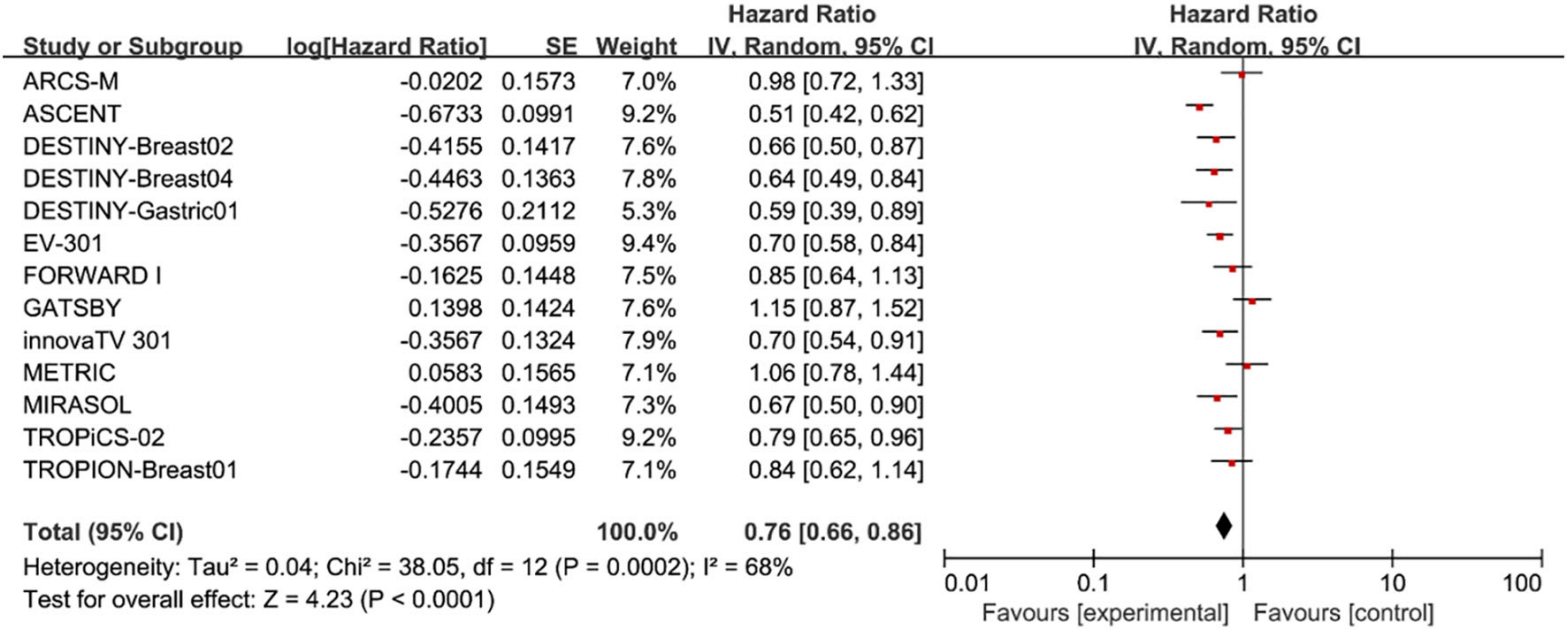
Forest plots comparing overall survival (OS) hazard ratios between treatment and control groups.

In terms of OS, we found that patients treated with ADC drugs exhibited superior outcomes compared to those treated with chemotherapy drugs (HR = 0.76; 95% CI: 0.66-0.86, p < 0.0001, **Figure 2**). A random effects model was used (I² = 68%).

### 3.5 Progression-free survival

**Figure 3:**
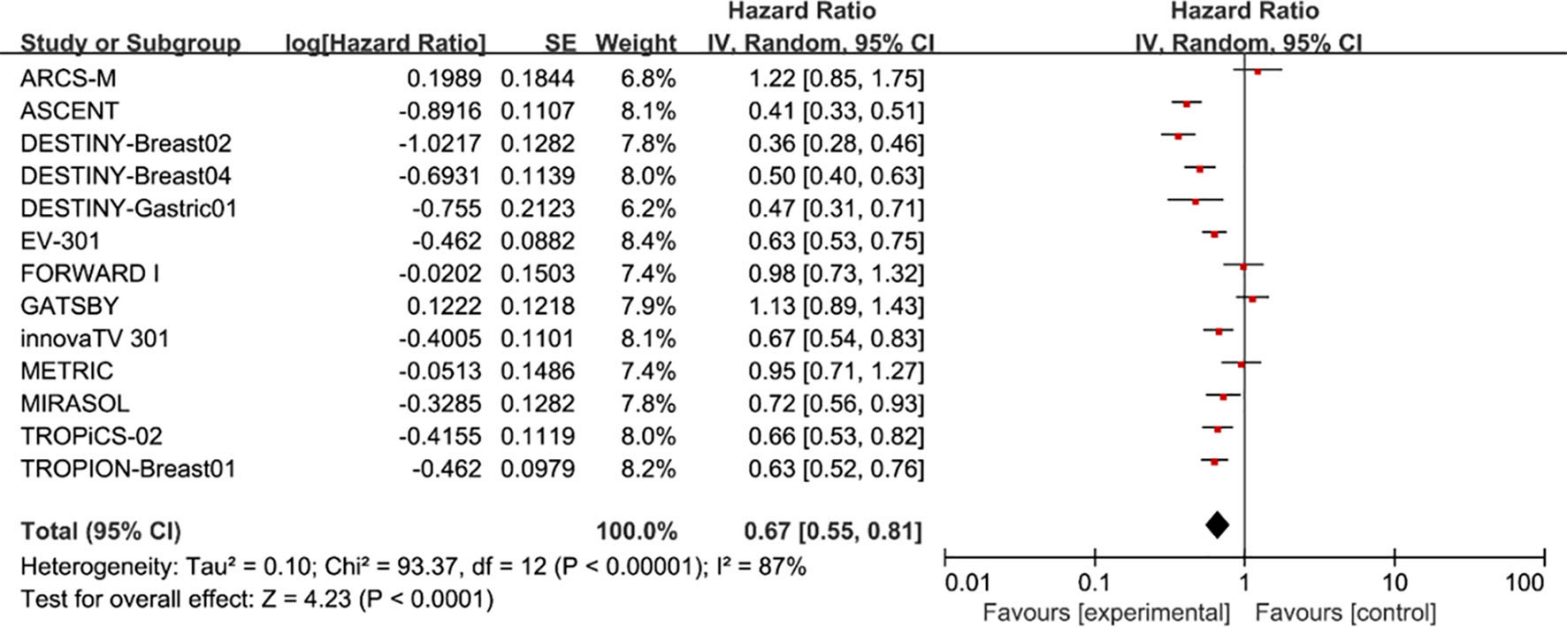
Forest plots comparing progression-free survival (PFS) hazard ratios between treatment and control groups.

Pooled analysis of selected cases showed that PFS in the experimental group was improved (HR = 0.67, 95%CI: 0.55-0.81; p < 0.0001, **Figure 3**), using a random effects model (I^2^ = 87%).

### 3.6 Selected subgroup analysis

**Figure 4:**
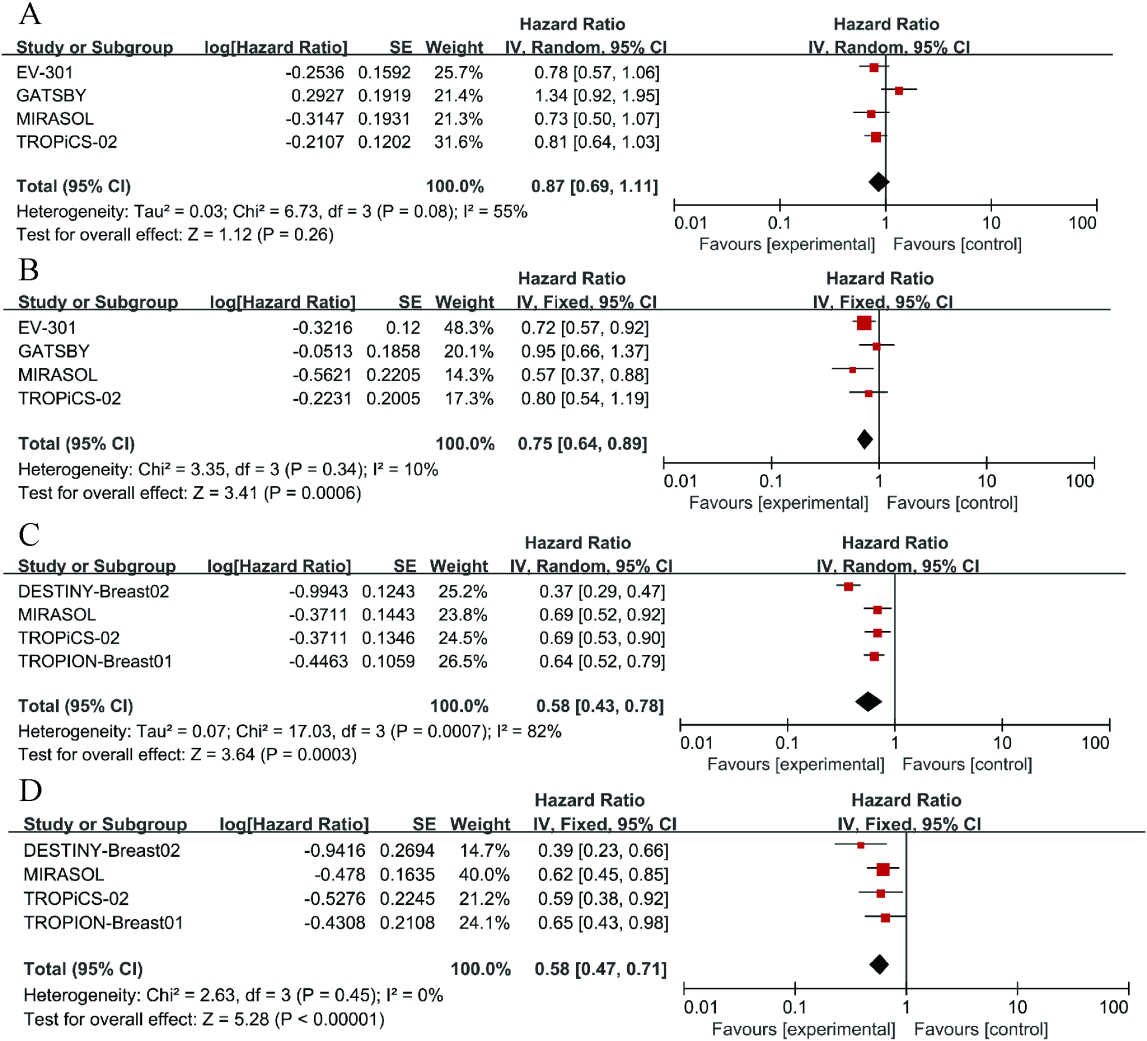
Forest plots comparing overall survival (OS) and progression-free survival (PFS) hazard ratios between ADC drugs and chemotherapy drugs. **(A)** The OS situation of patients under 65 years old. **(B)** The OS situation of patients over 65 years old. **(C)** The PFS situation of patients under 65 years old. **(D)** The PFS situation of patients over 65 years old.

**Figure 5:**
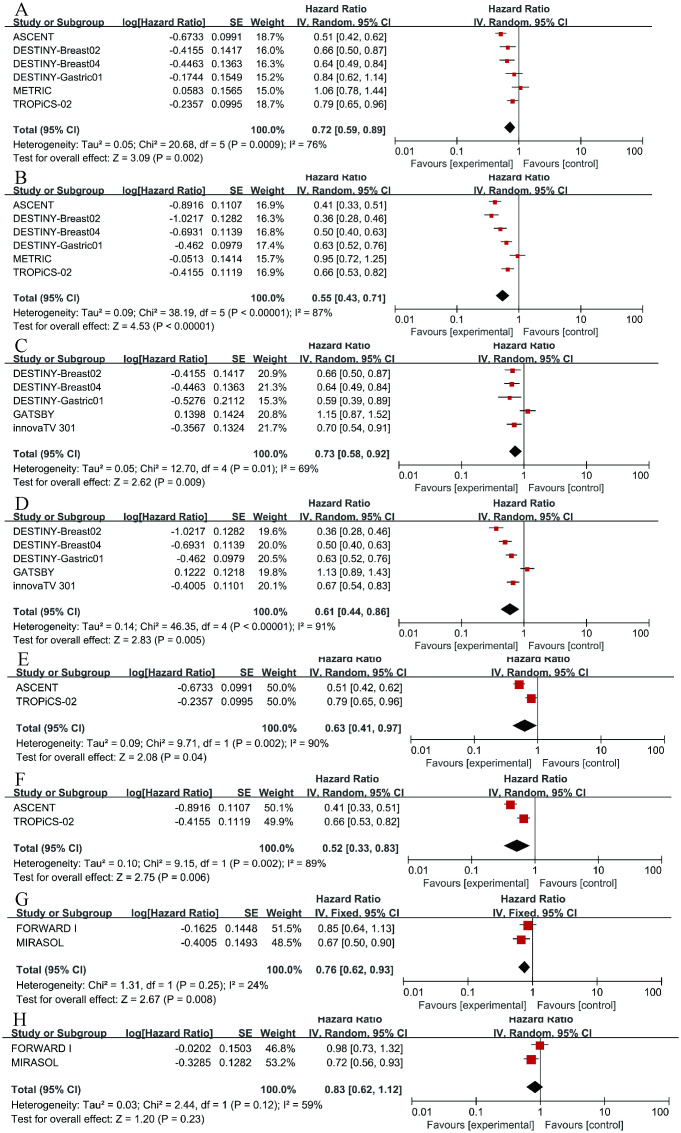
Forest plots comparing overall survival (OS) and progression-free survival (PFS) hazard ratios between ADC drugs and chemotherapy drugs. **(A)** OS in breast cancer. **(B)** PFS in breast cancer. **(C)** OS in Trastuzumab deruxtecan (T-DM1). **(D)** PFS in T-DM1. **(E)** OS in Sacituzumab govitecan (SG). **(F)** PFS in SG. **(G)** OS in Mirvetuximab soravtansine (Elahere). **(H)** PFS in Elahere.

To further examine the heterogeneity of the data, subgroup analyses were conducted based on cancer type and ADC drug type, as illustrated in **Figure 4**. The results indicate that in each subgroup analysis, ADC drugs exhibited superior efficacy compared to chemotherapy drugs. However, heterogeneity in differences remained. Among breast cancer patients, ADC drugs demonstrated higher efficacy in terms of PFS (HR = 0.55, 95%CI: 0.43-0.71; p < 0.0001, **Figure 5B**) compared to OS (HR = 0.72, 95%CI: 0.59-0.89; p = 0.002, **Figure 5A**). When Trastuzumab deruxtecan (T-DM1) was compared with chemotherapy drugs, patients benefited more in PFS (HR = 0.61, 95%CI: 0.44-0.86; p = 0.005, **Figure 5D**) than in OS (HR = 0.73, 95%CI: 0.58-0.92; p = 0.009, **Figure 5C**). Similar results were also observed for the drug Sacituzumab govitecan (SG), where PFS (HR = 0.52, 95% CI: 0.33-0.83; p = 0.006, **Figure 5F**) demonstrated a greater benefit compared to OS (HR = 0.63, 95% CI: 0.41-0.97; p = 0.004, **Figure 5E**). Due to I² > 50, a random-effects model was used for all the above analyses. In the comparison between Mirvetuximab soravtansine (Elahere) and chemotherapy, patients in the Elahere group had a better OS (HR = 0.63, 95% CI: 0.62-0.93; p = 0.008, **Figure 5G**) than those in the chemotherapy group, analyzed using a fixed-effects model (I² = 24%). However, the statistical result for PFS was not significant, with P = 0.23. Furthermore, subgroup analysis by age was also conducted. Compared with chemotherapy, the survival advantage of ADCs remained consistent across different age groups (**Figure 4**). For patients under 65 years old, no statistically significant improvement was observed in OS (P = 0.26, **Figure 4A**); nevertheless, PFS showed significant improvement (HR = 0.75, 95% CI: 0.64–0.89; p = 0.0006, I² = 10%, **Figure 4B**). Similarly, among patients aged 65 years or older, significant benefits were observed in both OS (HR = 0.58, 95% CI: 0.43–0.78; p = 0.0003, I² = 82%, **Figure 4C**) and PFS (HR = 0.58, 95% CI: 0.47–0.71; p < 0.00001, I² = 0%, **Figure 4D**). These results suggest that the efficacy advantage of ADCs is maintained irrespective of patient age.

### 3.7 Sensitivity analysis and publication bias

We conducted a sensitivity analysis based on the statistical results. After converting the random effects model to a fixed effects model, there were no significant changes in the results (**Supplementary Table S1**). The comprehensive risk of the primary outcome showed minimal variation after excluding individual trials, indicating the relative stability of the results (**Supplementary Tables S2**, **S3**). The GRADE assessment was conducted for the primary outcomes, OS and PFS (**Supplementary Table S4**), in order to enhance the reliability of the results.

### 3.8 Safety

**Figure 6:**
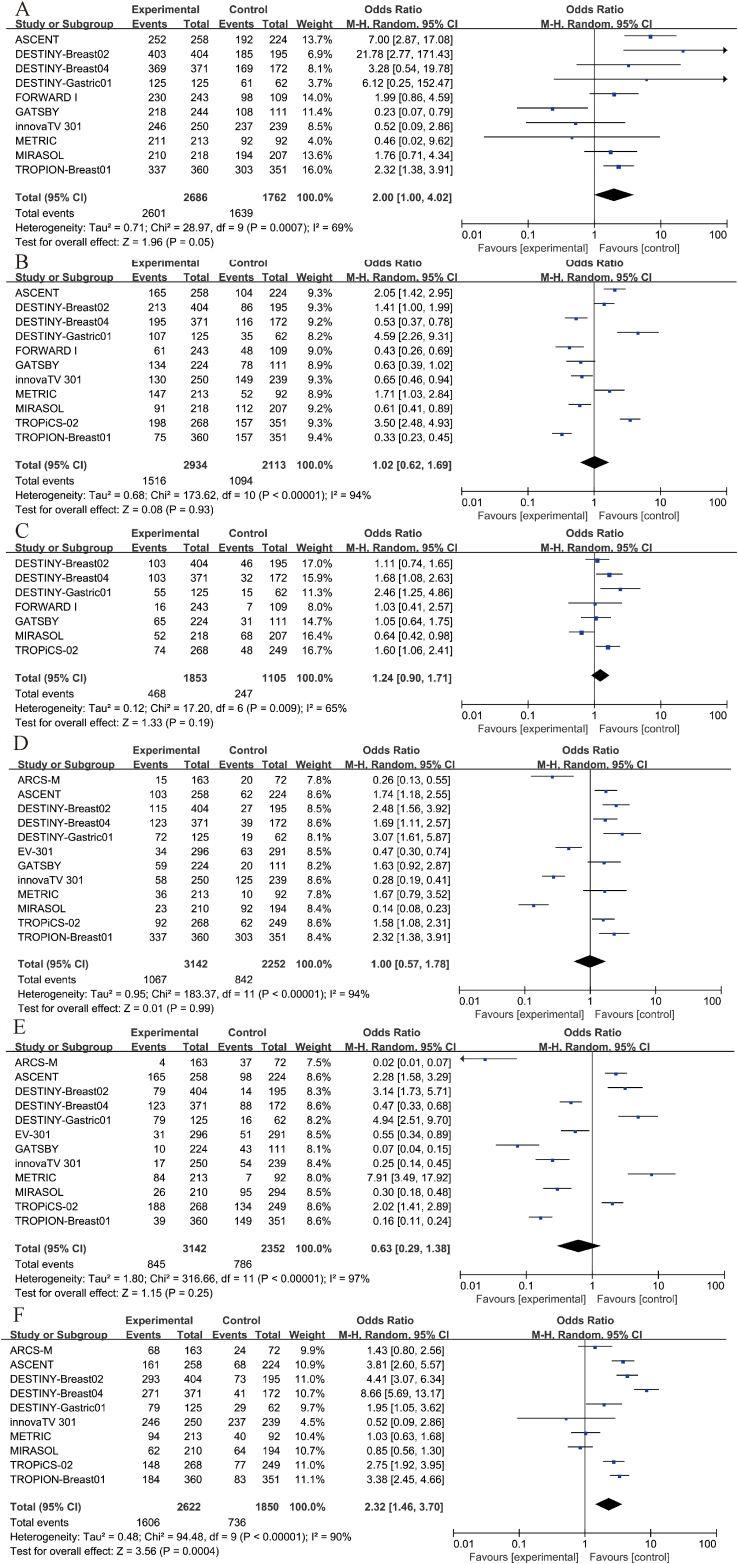
Security analysis. **(A)** Any adverse event results of ADC drugs and chemotherapy drugs. **(B)** Grade 3–5 AEs results of ADC drugs and chemotherapy drugs. **(C)** Severe AEs results of ADC drugs and chemotherapy drugs. **(D)** anemia results of ADC drugs and chemotherapy drugs. **(E)** neutropenia results of ADC drugs and chemotherapy drugs. **(F)** nausea results of ADC drugs and chemotherapy drugs.

Among patients receiving ADC therapy, 96.8% experienced adverse events of any grade, compared to 93.0% of those receiving chemotherapy. For adverse events of grade 3 or higher, 51.7% of patients in the ADC group and 51.8% in the chemotherapy group were affected. The incidence of serious adverse events was 25.3% in the ADC group and 22.4% in the chemotherapy group. However, none of these differences were considered significant (**Figures 6A–C**).

Furthermore, we conducted separate analyses for the most frequent specific adverse events, including anemia, neutropenia, and nausea. The results indicated that neither anemia nor neutropenia showed a statistically significant difference between groups (P > 0.05, **Figures 6D, E**). However, the incidence of nausea was significantly higher in the treatment group compared to the control group (OR = 2.32, 95% CI: 1.46–3.70; p = 0.0004, **Figure 6F**). Due to I² = 90%, a random-effects model was applied for this outcome.

## 4 Discussion

In the current database, we conducted a meta-analysis of thirteen RCT studies. To our knowledge, this is the first comprehensive meta-analysis comparing the efficacy of ADC drugs with that of chemotherapeutic agents. In our analysis, ADCs demonstrated superior efficacy compared to chemotherapeutic drugs in terms of both OS (HR = 0.67, 95%CI: 0.55-0.81) and PFS (HR = 0.76; 95% CI: 0.66-0.86). These hazard ratios translate to substantial and clinically meaningful survival benefits for patients. Specifically, a hazard ratio of 0.67 for OS corresponds to a 33% reduction in the risk of death, which, in practical terms, could represent an extension of median overall survival by several months, a significant gain in the context of advanced malignancies. Similarly, the HR of 0.76 for PFS indicates a 24% reduction in the risk of disease progression or death, which likely translates to a clinically important prolongation in the time patients live without their cancer worsening, potentially doubling the median PFS in some sensitive tumor types. Although some clinical study results indicate that ADC (Antibody-Drug Conjugate) drugs do not significantly improve disease progression in clinical patients (36), the majority of clinical study results support ADCs as having higher therapeutic efficacy. For instance, SG has been shown to improve OS in patients who have previously received treatment (37), and Brentuximab vedotin demonstrates favorable therapeutic effects in patients with Hodgkin lymphoma (38), among others. These findings align with our conclusions. This may be attributed to the more precise efficacy of ADC drugs, which can specifically recognize and bind to particular antigens on the surface of tumor cells through monoclonal antibodies, followed by endocytosis by the tumor cells. Inside the tumor cells, the linker is enzymatically cleaved, releasing the small molecule drug to exert its cytotoxicity (3). Consequently, most scholars believe that ADC drugs can broaden the therapeutic window and improve the effectiveness and safety of cancer treatment (39). With technological advancements and developments, an increasing number of ADC drugs have been applied clinically and have achieved favorable therapeutic outcomes (40). The development of ADCs frequently employs computational methods for target and compound optimization, a strategy that echoes the bioactivity modeling of natural products targeting viral proteases or oncogenic dimers (41, 42). However, there are also researchers who argue that ADC drugs may represent an upgraded version of chemotherapy drugs (43). Although their mechanism of action relies on antigen-antibody binding, this may simultaneously trigger potential immune resistance reactions, leading to the occurrence of adverse effects. Besides, despite continuous progress in the ADC field, the safety profile and toxicity mechanisms remain relatively obscure, with targeted/off-target toxicity and payload side effects potentially inducing related adverse event (44).

Chemotherapy is currently recognized as one of the more effective methods for treating tumors, based on the principle of using chemical drugs to kill or inhibit the growth of tumor cells (45). It is worth reflecting that the investigation of natural compounds and traditional medicines remains a valuable source of insight for modern therapeutics, as highlighted by systematic reviews of herbal remedies (46). Due to its significant therapeutic effects on most tumors, its applicability to tumor treatment at any stage, and its use as adjuvant therapy after surgery or radiotherapy, chemotherapy occupies an important position in tumor treatment (47). However, chemotherapy drugs also have the same killing effect on normal cells, resulting in varying degrees of adverse event in most patients, with severe cases leading to drug discontinuation (48). Additionally, some patients may develop drug resistance, which is also an important factor affecting treatment efficacy. Chemotherapy resistance is generally classified into primary resistance, acquired resistance, cross-resistance, and complete drug resistance, indicating that the causes of chemotherapy resistance are diverse (49). Once resistance develops, patients can only switch to alternative treatment regimens, making the research on new anticancer drugs and drug targets of great significance (50).

Due to the high heterogeneity observed in both OS and PFS in the analysis results, we conducted corresponding subgroup analyses. Firstly, we analyzed breast cancer, finding that ADCs improved patients’ OS (HR = 0.72, 95%CI: 0.59-0.89) and PFS (HR = 0.55, 95%CI: 0.43-0.71) compared to chemotherapy, with a more significant effect on OS. This finding aligns with previously published views, such as the strong antitumor activity of T-DM1 in treating HER2-positive breast cancer patients (51); ADCs have great potential in treating solid tumors, particularly in extending survival time for patients with HER2 IHC 3+ mutations (52). However, our statistical results still exhibited high heterogeneity, which may be attributed to inconsistencies in the chemotherapy agents, ADC drugs, and doses used. Additionally, T-DM1 (OS: HR = 0.73, 95%CI: 0.58-0.92; PFS: HR = 0.61, 95%CI: 0.44-0.86) and SG (OS: HR = 0.63, 95% CI: 0.41-0.97; PFS: HR = 0.52, 95% CI: 0.33-0.83) both showed benefits in OS and PFS compared to chemotherapy drugs, with a greater benefit observed in PFS. Several studies have indicated that T-DM1 can also exhibit favorable therapeutic effects in patients with HER2-mutant non-small cell lung cancer (53); SG has been beneficial for patients with breast cancer with brain metastases and recurrent glioblastoma (54); These findings are similar to our conclusions. In the research on Mirvetuximab soravtansine, compared to chemotherapy drugs, patients exhibited an improved OS (HR = 0.63, 95% CI: 0.62-0.93), although the improvement in PFS was not statistically significant (P = 0.23).

In terms of safety, although our analysis did not reach statistical significance (P > 0.05), the proportions of patients experiencing any adverse events (96.8% vs. 93.6%), grade 3–5 adverse event (51.7% vs. 51.8%), and serious adverse event (25.3% vs. 22.4%) were similar between ADC therapy and chemotherapy. A meta-analysis of numerous clinical trials indicated that the proportion of any adverse events associated with ADC-related treatment was 91.2%, and different ADCs seemed to influence the various adverse events related to their use (38). Overall, despite the fact that ADC drugs can cause adverse event in the majority of patients and pose a risk of continued medication, their safety profile remains acceptable (52). Our findings underscore the need for further well-designed, head-to-head RCTs that directly compare ADC therapy with standard chemotherapy regimens, especially within specific cancer subtypes and molecular contexts. Future studies should also focus on identifying predictive biomarkers, such as tumor antigen expression levels and mechanisms of drug resistance, to better stratify patients who are most likely to benefit from ADCs. In addition, more research is needed to evaluate the economic impact and cost-effectiveness of ADCs relative to conventional chemotherapy, which will be crucial for guiding clinical and policy decisions.

This study has several limitations. First, as our research is based on literature retrieval, there may be certain biases in the statistical results. Second, the medications and dosages administered for ADC drugs or chemotherapeutic agents vary among different experiments, and patients of different body sizes have different levels of tolerance to drug dosages, which may impose certain limitations on the experimental results. Additionally, the statistical results exhibit moderate to high heterogeneity, which may affect the credibility of the analysis. However, we have conducted the necessary sensitivity analysis and subgroup analysis to minimize the impact of heterogeneity on the statistical results. Finally, a key limitation lies in the potential overinterpretation of ADC superiority without adequate critical appraisal of variations in study quality, clinical context, and confounding factors. Parameters such as baseline patient characteristics (e.g., heavily pretreated vs. newly diagnosed populations), differences in trial design and endpoint definitions, and the impact of small sample sizes within certain subgroups were not thoroughly discussed. These omissions may limit the contextualization and generalizability of our findings.

## 5 Conclusion

In conclusion, this meta-analysis indicates that ADCs provide significantly improved PFS and OS compared to conventional chemotherapy, while maintaining a generally acceptable safety profile in several malignant tumors. However, these benefits must be interpreted with caution due to significant heterogeneity across studies and variations in patient baseline characteristics. Our results support the use of ADCs as a valuable therapeutic option, particularly in refractory or relapsed settings. Further studies are warranted to refine patient selection, optimize combination strategies, and clarify long-term outcomes.

## Data Availability

The original contributions presented in the study are included in the article/**Supplementary Material**. Further inquiries can be directed to the corresponding authors.
