# Supplementary material for: Comparative efficacy of antibody-drug conjugates and chemotherapy for malignant tumors: a systematic review and meta-analysis
Source: Front Oncol. 2026 Jan 9;15:1697340. doi: 10.3389/fonc.2025.1697340 (PMC12827071; doi:10.3389/fonc.2025.1697340)
Supplement: Supplementary file 1 [file DataSheet1.docx]

**Supplementary Method 1** - Search strategy for PubMed

((Neoplasms [MeSH Terms] OR (neoplas* [Title/Abstract] OR tumor* [Title/Abstract] OR cancer* [Title/Abstract] OR malignan* [Title/Abstract] OR tumour* [Title/Abstract] OR carcinom* [Title/Abstract] OR adenocarcin* [Title/Abstract] ) ) AND (Immunoconjugates [MeSH Terms]) OR ((Antibody-Drug Conjugates [Title/Abstract]) OR (adc [Title/Abstract]) OR (Brentuximab Vedotin [Title/Abstract]) OR (Adcetris [Title/Abstract]) OR (SGN-35 [Title/Abstract]) OR (Belantamab Mafodotin [Title/Abstract]) OR (Blenrep [Title/Abstract]) OR (GSK2857916 [Title/Abstract]) OR (Cetuximab Saratolacan [Title/Abstract]) OR (Akalux [Title/Abstract]) OR (Enfortumab Vedotin [Title/Abstract]) OR (Padcev [Title/Abstract]) OR (ASG-22ME [Title/Abstract]) OR (Gemtuzumab Ozogamicin [Title/Abstract]) OR (Mylotarg [Title/Abstract]) OR (CMA-676 [Title/Abstract]) OR (Inotuzumab Ozogamicin [Title/Abstract]) OR (Besponsa [Title/Abstract]) OR (CMC-544 [Title/Abstract]) OR (Moxetumomab Pasudotox [Title/Abstract]) OR (Lumoxiti [Title/Abstract]) OR (Polatuzumab Vedotin [Title/Abstract]) OR (Polivy [Title/Abstract]) OR (Sacituzumab Govitecan [Title/Abstract]) OR (Trodelvy [Title/Abstract]) OR (IMMU-132 [Title/Abstract]) OR (Trastuzumab Deruxtecan [Title/Abstract]) OR (Enhertu [Title/Abstract]) OR (DS-8201 [Title/Abstract]) OR (Trastuzumab Emtansine [Title/Abstract]) OR (Kadcyla [Title/Abstract]) OR (TDM-1 [Title/Abstract]) OR (1959-sss-DM3 [Title/Abstract]) OR (A166 [Title/Abstract]) OR (ABBV-085 [Title/Abstract]) OR (ABBV-321 [Title/Abstract]) OR (ABBV-399 [Title/Abstract]) OR (ADCT-301 [Title/Abstract]) OR (ADCT-402 [Title/Abstract]) OR (A-dmDT390-bisFv [Title/Abstract]) OR (Anetumab ravtansine [Title/Abstract]) OR (ARX788 [Title/Abstract]) OR (ATOR-1015 [Title/Abstract]) OR (B003 [Title/Abstract]) OR (BAT8001 [Title/Abstract]) OR (BAY 94-9343 [Title/Abstract]) OR (BAY-943 [Title/Abstract]) OR (BMS-986148 [Title/Abstract]) OR (BT-062 [Title/Abstract]) OR (BT1718 [Title/Abstract]) OR (BT5528 [Title/Abstract]) OR (Camidanlumab tesirine [Title/Abstract]) OR (cofetuzumab pelidotin [Title/Abstract]) OR (CX-2009 [Title/Abstract]) OR (CX-2029 [Title/Abstract]) OR (Datopotamab deruxtecan [Title/Abstract]) OR (depatuxizumab mafodotin [Title/Abstract]) OR (disitamab vedotin [Title/Abstract]) OR (Dolaflexin [Title/Abstract]) OR (DS-1062a [Title/Abstract]) OR (DS-6157a [Title/Abstract]) OR (Enapotamab vedotin [Title/Abstract]) OR (F0002-ADC [Title/Abstract]) OR (HuMax-TF-ADC [Title/Abstract]) OR (IMGN-853 [Title/Abstract]) OR (Indatuximab ravtansine [Title/Abstract]) OR (Ladiratuzumab vedotin [Title/Abstract]) OR (L-DOS47 [Title/Abstract]) OR (Loncastuximab tesirine [Title/Abstract]) OR (Lorvotuzumab Mertansine [Title/Abstract]) OR (MEDI2228 [Title/Abstract]) OR (MEN1309 [Title/Abstract]) OR (MGC018 [Title/Abstract]) OR (Mirvetuximab soravtansine [Title/Abstract]) OR (Mitazalimab [Title/Abstract]) OR (MORAb-202 [Title/Abstract]) OR (Naratuximab emtansine [Title/Abstract]) OR (OBI-999 [Title/Abstract]) OR (Oportuzumab Monatox [Title/Abstract]) OR (patritumab deruxtecan [Title/Abstract]) OR (PF-06647020 [Title/Abstract]) OR (PF-06804103 [Title/Abstract]) OR (PSMA-ADC [Title/Abstract]) OR (RC48-ADC [Title/Abstract]) OR (SAR408701 [Title/Abstract]) OR (Serclutamab talirine [Title/Abstract]) OR (SGN-LIV1A [Title/Abstract]) OR (SHR-A1403 [Title/Abstract]) OR (STRO-001 [Title/Abstract]) OR (SYD985 [Title/Abstract]) OR (TAK-164 [Title/Abstract]) OR (telisotuzumab vedotin [Title/Abstract]) OR (Tisotumab vedotin [Title/Abstract]) OR (Trastuzumab duocarmazine [Title/Abstract]) OR (U3-1402 [Title/Abstract]) OR (XMT-1536 [Title/Abstract]) OR (XMT-1592 [Title/Abstract])) AND ((((((((randomized controlled trial[Publication Type]) OR controlled clinical trial[Publication Type]) OR randomized[Title/Abstract]) OR placebo[Title/Abstract]) OR randomly[Title/Abstract]) OR "Clinical Trials as Topic"[Mesh:NoExp]) OR trial[Title])) NOT ((((animals[MeSH Terms]) NOT ((humans[MeSH Terms]) AND animals[MeSH Terms]))))) AND ((chemotherapy[MeSH Terms]) OR (Therapy, Drug OR Drug Therapies OR Therapies, Drug OR Chemotherapy OR Chemotherapies OR Pharmacotherapy OR Pharmacotherapies))

**Supplementary Method 2** - Search strategy for Cochrane

#1 MeSH descriptor: [Immunoconjugates] explode all trees

#2 (Antibody Drug Conjugates):ab,ti,kw OR (ADC):ab,ti,kw OR (Brentuximab Vedotin):ab,ti,kw OR (Adcetris):ab,ti,kw OR (SGN-35):ab,ti,kw OR (Belantamab Mafodotin):ab,ti,kw OR (Blenrep):ab,ti,kw OR (GSK2857916):ab,ti,kw OR (Cetuximab Saratolacan):ab,ti,kw OR (Akalux):ab,ti,kw OR (Enfortumab Vedotin):ab,ti,kw OR (Padcev):ab,ti,kw OR (ASG-22ME):ab,ti,kw OR (gemtuzumab ozogamici):ab,ti,kw

#3 (Mylotarg):ab,ti,kw OR (CMA-676):ab,ti,kw OR (inotuzumab ozogamici):ab,ti,kw OR (Besponsa):ab,ti,kw OR (CMC-544):ab,ti,kw OR (Moxetumomab Pasudotox):ab,ti,kw OR (Lumoxit):ab,ti,kw OR (Polatuzumab Vedotin):ab,ti,kw OR (Polivy):ab,ti,kw OR (Sacituzumab Govitecan):ab,ti,kw OR (Trodelvy):ab,ti,kw OR (IMMU-132):ab,ti,kw

#4 (Trastuzumab Deruxtecan):ab,ti,kw OR (Enhertu):ab,ti,kw OR (DS-8201):ab,ti,kw OR (Trastuzumab Emtansine):ab,ti,kw OR (Kadcyla):ab,ti,kw OR (TDM-1):ab,ti,kw

#5 #1 OR #2 OR #3 OR #4

#6 MeSH descriptor: [Neoplasms] explode all trees

#7 (neoplas*):ti,ab,kw OR (tumor*):ti,ab,kw OR (cancer*):ti,ab,kw OR (malignan*):ti,ab,kw OR (tumour*):ti,ab,kw

#8 (carcinom*):ti,ab,kw OR (adenocarcin*):ti,ab,kw

#9 #6 OR #7 OR #8

#10 (Therapy, Drug*):ti,ab,kw OR (Drug Therapies*):ti,ab,kw OR (Therapies*):ti,ab,kw OR (Chemotherapy*):ti,ab,kw OR (Chemotherapies*):ti,ab,kw OR (Pharmacotherapy *):ti,ab,kw OR (Pharmacotherapies *):ti,ab,kw

#11 #5 AND #9 AND #10

**Supplementary Method 3** - Search strategy for Embase

#1 AND #2 AND #3 AND #4

1,426

#4

'randomized controlled trial'/exp OR 'randomization'/exp OR 'double blind procedure'/exp OR 'placebo'/exp

1,228,287

#3

'chemotherapy'/exp OR 'therapy, drug'/exp OR 'pharmacotherapy'/exp

3,861,626

#2

'antibody conjugate'/exp OR 'antibody-drug conjugates':ab,ti OR adc:ab,ti OR 'brentuximab vedotin':ab,ti OR adcetris:ab,ti OR 'sgn 35':ab,ti OR 'belantamab mafodotin':ab,ti OR blenrep:ab,ti OR gsk2857916:ab,ti OR 'cetuximab saratolacan':ab,ti OR akalux:ab,ti OR 'enfortumab vedotin':ab,ti OR padcev:ab,ti OR 'asg 22me':ab,ti OR 'gemtuzumab ozogamicin':ab,ti OR mylotarg:ab,ti OR 'cma 676':ab,ti OR 'inotuzumab ozogamicin':ab,ti OR besponsa:ab,ti OR 'cmc 544':ab,ti OR 'moxetumomab pasudotox':ab,ti OR lumoxiti:ab,ti OR 'polatuzumab vedotin':ab,ti OR polivy:ab,ti OR 'sacituzumab govitecan':ab,ti OR trodelvy:ab,ti OR 'immu 132':ab,ti OR 'trastuzumab deruxtecan':ab,ti OR enhertu:ab,ti OR 'ds 8201':ab,ti OR 'trastuzumab emtansine':ab,ti OR kadcyla:ab,ti OR 'tdm 1':ab,ti

52,873

#1

'neoplasm'/exp OR neoplas*:ab,ti OR tumor*:ab,ti OR cancer*:ab,ti OR malignan*:ab,ti OR tumour*:ab,ti OR carcinom*:ab,ti OR adenocarcin*:ab,ti

7,560,137


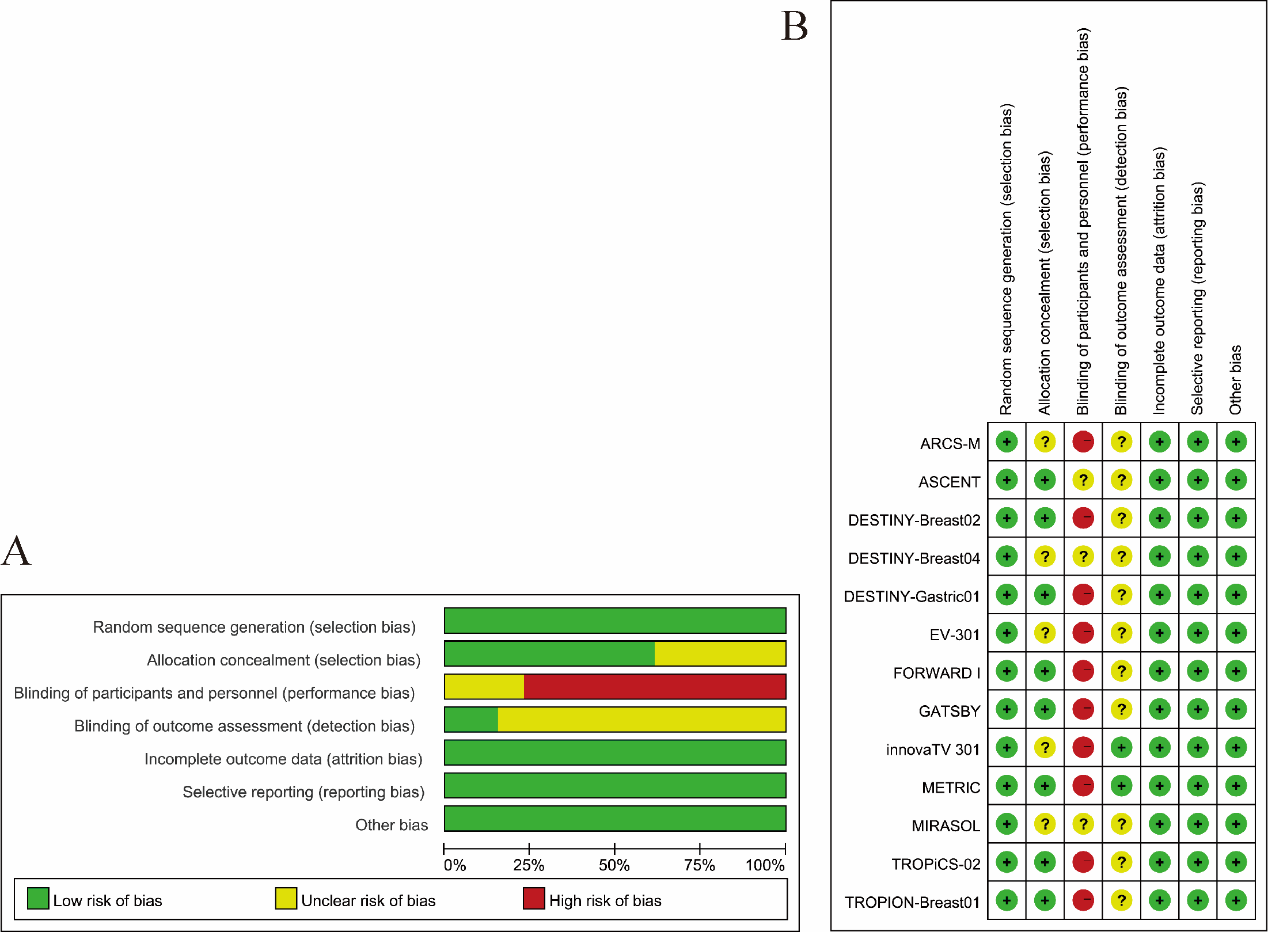


**Supplementary Figure 1**

Risk of bias of included studies. (A): Risk of bias graph. (B): Risk of bias summary: Assessment of the nine included studies for risk of bias items (" + ": low risk of bias; " ? ": unclear risk of bias; " − "：high risk of bias")


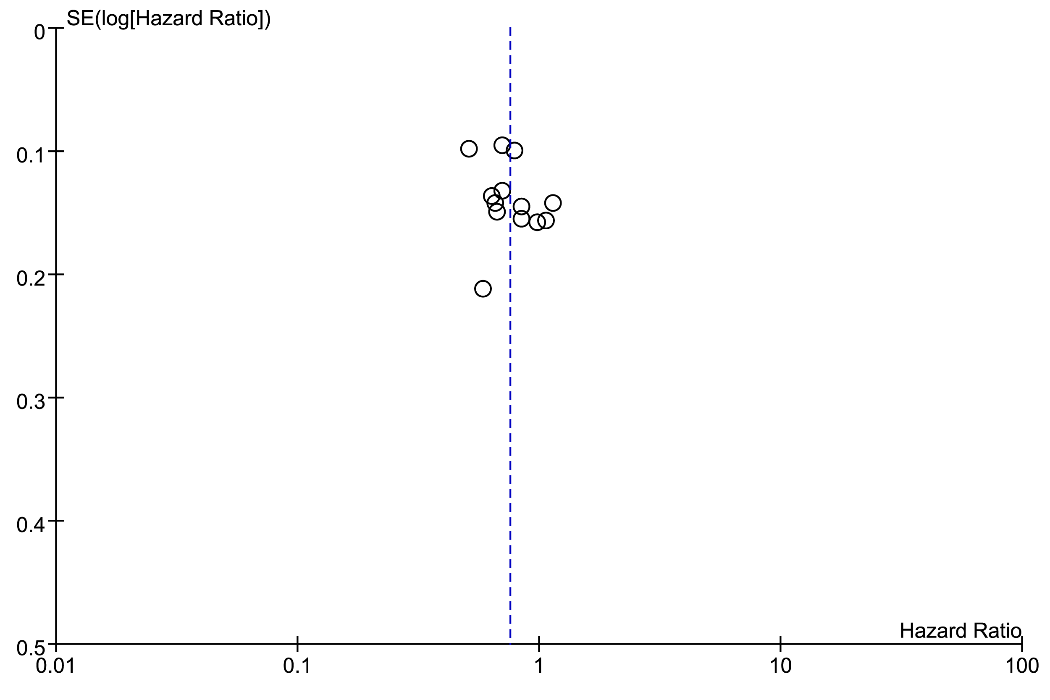


**Supplementary Figure 2**

Funnel plot of the OS correlation between the treatment patients and the control group
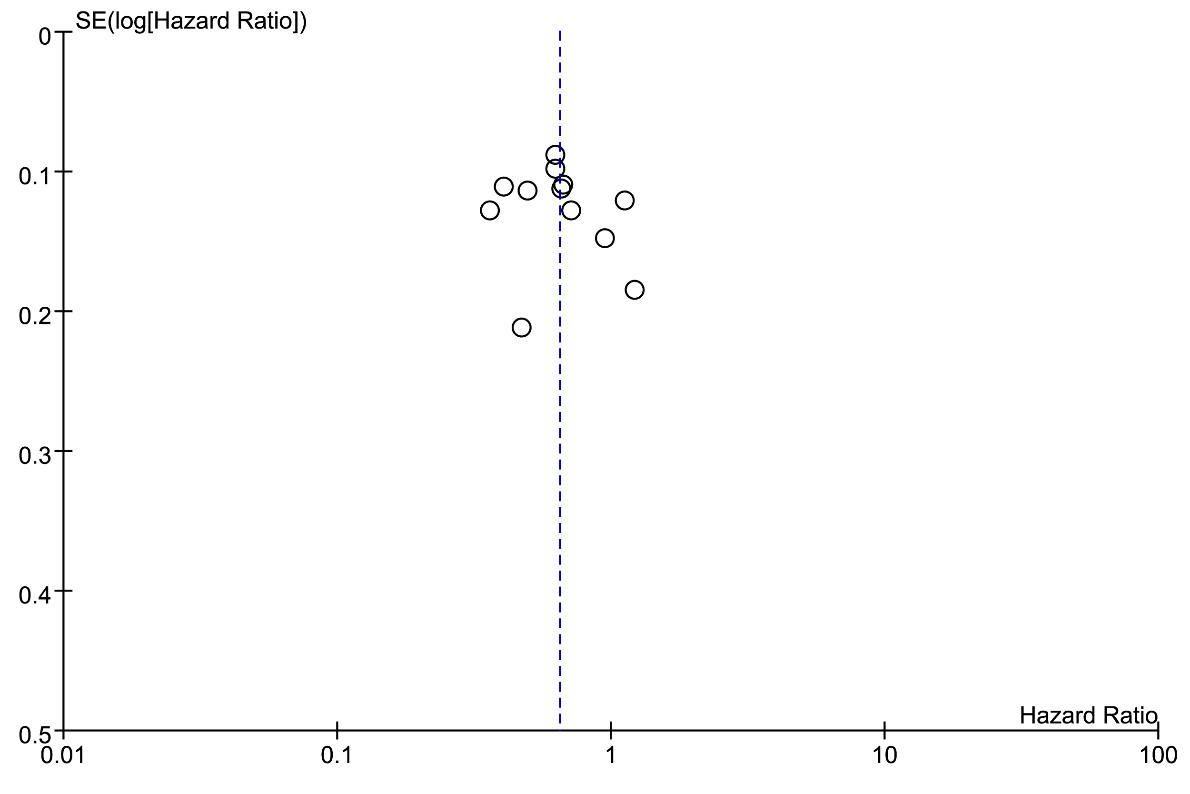


**Supplementary Figure 3**

Funnel plot of the PFS correlation between the treatment patients and the control group

**Supplementary Table S1. Sensitivity analysis of studies included**

| Excluded study | Hazard ratio | 95% CI | | *I*² | *P* |
| --- | --- | --- | --- | --- | --- |
| / | 0.76 | 0.66-0.86 | 68% | | *P* < 0.0001 |
| ARCS-M | 0.74 | 0.65-0.85 | 68% | | *P* < 0.00001 |
| METRIC | 0.74 | 0.65-0.84 | 66% | | *P* < 0.00001 |
| GATSBY | 0.73 | 0.65-0.82 | 60% | | *P* < 0.00001 |
| MIRASOL | 0.76 | 0.66-0.88 | 71% | | *P* = 0.0001 |
| innovaTV 301 | 0.76 | 0.66-0.88 | 71% | | *P* = 0.0001 |
| DESTINY-Gastric01 | 0.77 | 0.67-0.88 | 70% | | *P* = 0.0001 |
| DESTINY-Breast02 | 0.76 | 0.66-0.88 | 71% | | *P* = 0.0002 |
| DESTINY-Breast04 | 0.77 | 0.67-0.88 | 70% | | *P* = 0.0002 |
| ASCENT | 0.79 | 0.70-0.88 | 50% | | *P* < 0.0001 |
| TROPION-Breast01 | 0.75 | 0.65-0.86 | 70% | | *P* = 0.0001 |
| TROPiCS-02 | 0.75 | 0.65-0.87 | 71% | | *P* = 0.0001 |
| EV-301 | 0.76 | 0.66-0.88 | 71% | | *P* = 0.0002 |
| FORWARD I | 0.75 | 0.65-0.86 | 70% | | *P* < 0.0001 |

Notes: PFS/OS: HR, 95%CI (Treatment group vs. control group); Any adverse event/ Grades 3-5 AEs/ Severe AEs: RR, 95%CI (Treatment group vs. control group).

**Supplementary Table S2. Sensitivity analysis regarding OS results of** **antibody-drug conjugate and chemotherapy**

|  | **Random effect model** | **Fixed effect model** |
| --- | --- | --- |
| OS | 0.76 (0.66-0.86) | 0.74 (0.69-0.79) |
| PFS | 0.67 (0.55-0.81) | 0.64 (0.60-0.69) |
| OS (breast cancer) | \| 0.72 (0.59-0.89) \| \| --- \| | 0.69 (0.63-0.77) |
| PFS (breast cancer) | \| \| 0.55 (0.43-0.71) \| \| --- \| \| \| --- \| --- \| | 0.55 (0.50-0.60) |
| OS (trastuzumab deruxtecan) | \| 0.73 (0.58-0.92) \| \| --- \| | 0.74 (0.65-0.84) |
| PFS (trastuzumab deruxtecan) | \| 0.61 (0.44-0.86) \| \| --- \| | 0.62 (0.56-0.68) |
| OS (sacituzumab govitecan) | \| \| 0.63 (0.41-0.97) \| \| --- \| \| \| --- \| --- \| | 0.63 (0.55-0.73) |
| PFS (sacituzumab govitecan) | \| 0.52 (0.33-0.83) \| \| --- \| | 0.52 (0.44-0.61) |

**Supplementary Table S3. Sensitivity analysis regarding PFS results of antibody-drug conjugate and chemotherapy**

| Excluded study | Hazard ratio | 95% CI | | *I^2^* | *P* |
| --- | --- | --- | --- | --- | --- |
| / | 0.67 | 0.55-0.81 | 87% | | *P* < 0.0001 |
| ARCS-M | 0.64 | 0.53-0.77 | 86% | | *P* < 0.00001 |
| METRIC | 0.65 | 0.53-0.79 | 87% | | *P* < 0.0001 |
| GATSBY | 0.64 | 0.53-0.76 | 84% | | *P* < 0.00001 |
| MIRASOL | 0.66 | 0.54-0.81 | 88% | | *P* < 0.0001 |
| innovaTV 301 | 0.67 | 0.54-0.82 | 88% | | *P* < 0.0001 |
| DESTINY-Gastric01 | 0.68 | 0.56-0.83 | 88% | | *P* < 0.0001 |
| DESTINY-Breast02 | 0.70 | 0.59-0.84 | 85% | | *P* < 0.0001 |
| DESTINY-Breast04 | 0.68 | 0.56-0.84 | 87% | | *P* < 0.0002 |
| ASCENT | 0.70 | 0.58-0.84 | 85% | | *P* < 0.0001 |
| TROPION-Breast01 | 0.67 | 0.55-0.83 | 88% | | *P* < 0.00001 |
| TROPiCS-02 | 0.67 | 0.54-0.83 | 88% | | *P* < 0.00001 |
| EV-301 | 0.67 | 0.52-0.81 | 88% | | *P* < 0.0001 |
| FORWARD I | 0.65 | 0.53-0.78 | 87% | | *P* < 0.00001 |

**GRADE Assessment**

The GRADE assessment was conducted separately for the critical outcomes of OS and PFS. The process can be summarized in the following four steps:

1. Starting Evidence Level: All RCTs start as High quality.

2. Downgrading Factors: Five domains are considered for downgrading the evidence (-1 level for 'Serious', -2 for 'Very Serious'):

Risk of Bias: Are there serious methodological flaws in the included RCTs?

Inconsistency: Is there substantial variation in results across studies?

Imprecision: Is the confidence interval for the effect estimate wide enough to cross a decision-making threshold?

Indirectness: Does the evidence directly answer the PICO question?

Publication Bias: Is there suspicion of unpublished negative results?

3. Upgrading Factors: Three domains are considered for upgrading the evidence:

Large Magnitude of Effect: An upgrade may be considered if HR < 0.5 or > 2.

Dose-Response Gradient: Not applicable.

Plausible Confounding: Not applicable.

4. Final Evidence Level: Integrating the above, the final evidence quality is categorized into one of four grades: High, Moderate, Low, or Very Low.

**Supplementary Table S4. The grading assessment form for OS and PFS**

| Outcome | Effect Estimate (95% CI) | Number of Studies (Participants) | Quality of the Evidence (Grade) | Reasons/Explanations |
| --- | --- | --- | --- | --- |
| OS | HR 0.76 (0.66-0.86) | 13 (5927) | ⊕⊕◯◯ Low | Reasons for Downgrading: 1. Risk of Bias (-1): Included studies were at high risk of selection bias. 2. Inconsistency (-1): High heterogeneity was observed (I² = 68%). |
| PFS | HR 0.67 (0.55-0.81) | 13 (5927) | ⊕⊕◯◯ Low | Reasons for Downgrading: 1. Risk of Bias (-1): Included studies were at high risk of selection bias. 2. Inconsistency (-1): Considerable heterogeneity was observed (I² = 87%). |
